# Biphasic Responses of Porcine Oocytes to Metformin: Concentration-Dependent AMPK Activation and Nrf2-Mediated Antioxidant Regulation

**DOI:** 10.3390/ani16121828

**Published:** 2026-06-13

**Authors:** Junyu Wang, Min Li, Yaqi Zhou, Fuyin Fu, Feng Liu, Jinghe Tan, Mingjiu Luo, Shuai Gong

**Affiliations:** 1 Shandong Provincial Key Laboratory for Livestock Germplasm Innovation & Utilization, College of Animal Science and Technology, Shandong Agricultural University, Tai’an 271018, China; 13370685817@163.com (J.W.); 18353879946@163.com (M.L.); 15275999891@163.com (Y.Z.); 15288916369@163.com (F.F.); tanjh@sdau.edu.cn (J.T.); luomj@sdau.edu.cn (M.L.); 2 Tai’an Bureau of Agriculture and Rural Affairs, Tai’an 271018, China; liufeng-p@163.com

**Keywords:** metformin, porcine oocyte, developmental competence, oxidative stress

## Abstract

Metformin (MET) plays crucial regulatory roles in mammalian oocyte meiosis, yet the concentration-dependent biphasic impacts of MET on porcine oocyte in vitro maturation (IVM) and the related molecular mechanisms remain poorly clarified. This study aimed to explore the distinct effects and underlying pathways of low- and high-dose MET in porcine oocytes. Different concentrations of MET (0, 7.5, 15, 30, 150, and 300 μM) were supplemented during oocyte IVM, with phenotypic detection, untargeted metabolomic analysis, and Nrf2 inhibitor (ML385) intervention performed for mechanism exploration. Results showed that 15 μM low-dose MET facilitated oocyte maturation, mitochondrial function and redox balance, while 300 μM high-dose MET caused obvious developmental damage. Mechanistically, low-dose MET triggered noncanonical AMPK activation independent of the AMP/ATP ratio and enhanced AMPK–Nrf2 antioxidant signaling, whereas high-dose MET induced energy stress and oxidative injury via inhibiting mitochondrial complex I. Blockade of Nrf2 further abolished the protective effects of low-dose MET. Collectively, this finding illustrates the biphasic actions of MET on porcine oocytes and provides a theoretical reference for optimizing porcine in vitro embryo production.

## Simple Summary

Poor porcine oocyte and embryo quality limits pig breeding efficiency. This study tested metformin supplementation in culture media to improve oocyte maturation and embryo development. Low-dose metformin enhanced maturation and blastocyst formation by activating AMPK through a non-canonical pathway, reducing oxidative damage and improving mitochondrial function, while high doses impaired oocyte quality. Appropriate metformin use optimizes in vitro culture systems, supporting more efficient pig embryo production for agriculture and reproductive research.

## 1. Introduction

Porcine oocyte in vitro maturation (IVM) is a pivotal technology with profound implications for both basic reproductive biology research and practical applications, including animal husbandry, germplasm conservation of endangered and elite breeds, and assisted reproductive technologies (ARTs) [1,2]. It serves as a critical link in the in vitro production (IVP) system of porcine embryos, laying the foundation for subsequent in vitro fertilization (IVF) and embryo culture (IVC) [2]. However, the developmental potential of porcine oocytes matured in vitro remains suboptimal compared to their in vivo counterparts, primarily due to inadequate cytoplasmic maturation and vulnerability to external stressors [3,4]. This limitation has become a bottleneck restricting the widespread application of porcine IVM technology, highlighting the urgent need to explore effective strategies for improving oocyte quality during IVM.

Oxidative stress stands out as a key detrimental factor affecting porcine oocyte IVM. During in vitro culture, the imbalance between reactive oxygen species (ROS) production and antioxidant defense mechanisms leads to the accumulation of ROS in oocytes [5]. Excessive ROS can induce oxidative damage to critical cellular components, including mitochondrial DNA, proteins, and lipids, thereby disrupting mitochondrial function, impairing cytoskeletal integrity, and interfering with intracellular signaling pathways involved in meiotic maturation and cytoplasmic remodeling [6,7]. These adverse effects ultimately result in reduced oocyte maturation rate, compromised fertilization capacity, and diminished embryonic developmental potential, making the mitigation of oxidative stress a crucial focus for optimizing IVM systems [8].

Metformin (1,1-dimethylbiguanide), a first-line therapeutic agent for type 2 diabetes mellitus (T2D), has garnered increasing attention beyond its hypoglycemic effects [9,10]. In recent years, accumulating evidence has demonstrated its efficacy in the treatment of polycystic ovary syndrome (PCOS), a common reproductive endocrine disorder associated with insulin resistance and impaired oocyte quality [11,12]. This clinical application has spurred the extension of metformin research into the field of reproduction and fertility, with studies exploring its potential protective roles in oocyte development. However, research on metformin’s effects on porcine oocytes remains relatively scarce, and a notable limitation of existing studies is the frequent use of suprapharmacological concentrations (exceeding 100 μM) [13,14,15], which are far higher than the clinically relevant plasma concentrations (5–40 μM) observed in patients receiving standard therapeutic doses (1.5–2 g/day) [16,17]. Such high concentrations may induce non-specific cellular responses that do not reflect the physiological mechanisms of metformin action, underscoring the necessity of investigating the effects of low, clinically relevant concentrations of metformin on porcine oocyte maturation and developmental competence. The 15 μM metformin used in this study falls within this physiological range, supporting its translational relevance.

Importantly, the mechanisms of action of metformin differ drastically between low and high concentrations. High concentrations of metformin are widely reported to inhibit mitochondrial complex I, leading to reduced ATP production, increased AMP/ATP ratios, and subsequent activation of the AMP-activated protein kinase (AMPK) pathway [18,19]. In contrast, low concentrations of metformin do not alter cellular adenine nucleotide levels (AMP/ATP) but instead exert their effects through distinct pathways, such as inhibiting the lysosomal v-ATPase via binding to PEN2 or regulating redox balance by inhibiting mitochondrial glycerophosphate dehydrogenase (GPD2) [20]. Previous studies have shown that metformin can improve the quality of vitrified-warmed porcine oocytes by alleviating cryoinjuries, and AMPK, as a master regulator of cellular energy metabolism and stress responses [21], plays a pivotal role in mediating metformin’s protective effects in various cell types [22]. However, the specific mechanisms by which low concentrations of metformin regulate porcine oocyte maturation, as well as whether AMPK signaling is involved, remain unclear.

Given the distinct functional targets of low versus high concentrations of metformin and the critical role of AMPK in oocyte physiology, systematic investigations comparing the effects of both concentration ranges on porcine oocytes are essential. Such studies not only contribute to a deeper understanding of metformin’s concentration-dependent mechanisms in reproductive cells but also provide valuable insights for the rational application of metformin to optimize porcine IVM systems and enhance oocyte quality. Therefore, this study aims to explore the mechanism by which low concentrations of metformin improve the maturation and developmental potential of porcine oocytes, with a focus on its interactions with oxidative stress and the AMPK signaling pathway, ultimately providing a theoretical basis for the clinical application of metformin in porcine reproductive biotechnology.

## 2. Materials and Methods

All porcine ovarian tissue samples used in this study were formally purchased from the Feicheng Slaughterhouse of Yinbao Food Corporation Ltd. (Tai’an, China). Meanwhile, all experimental protocols were reviewed and approved by the Animal Care and Use Committee of Shandong Agricultural University, China, on 10 March 2026 (Permit No. SDAUA-2026-103). Unless otherwise specified, all chemicals and reagents were purchased from Sigma-Aldrich Corp. (St. Louis, MO, USA).

### 2.1. Oocyte Collection, In Vitro Maturation and Drug Treatment

Porcine ovaries were collected from 6-month-old pigs and transported to the laboratory within 2 h in sterile physiological saline (30–35 °C), which was supplemented with 100 IU/mL penicillin and 0.05 mg/mL streptomycin. Cumulus–oocyte complexes (COCs) were recovered by aspirating 3–6 mm follicles using a syringe containing Dulbecco’s phosphate-buffered saline (D-PBS, HyClone, Logan, UT, USA) supplemented with 0.88 mM CaCl_2_·2H_2_O, 0.49 mM MgCl_2_·6H_2_O, 0.1% polyvinyl alcohol, 0.03 mM phenol red, 50 IU/mL penicillin, and 0.05 mg/mL streptomycin. Recovered COCs were washed three times in the same D-PBS solution, and only those with uniform ooplasm and surrounded by 3–5 layers of compact cumulus cells were selected for subsequent in vitro maturation.

Oocyte maturation was performed in TCM-199 medium (Gibco, Grand Island, NY, USA) supplemented with 0.91 mM sodium pyruvate, 4.0 mg/mL BSA, 3.05 mM D-glucose, 50 IU/mL penicillin, 0.05 mg/mL streptomycin, 0.05 IU/mL FSH, 0.05 IU/mL LH, 10 ng/mL EGF, and 0.57 mM cysteine. Maturation medium was aliquoted into 96-well plates (150 μL per well), overlaid with 100 μL mineral oil, and pre-equilibrated at 38.5 °C under 5% CO_2_ in humidified air for at least 3 h before use. After being washed three times in D-PBS and once in maturation medium, approximately 25 COCs per well were transferred into the pre-equilibrated wells, overlaid with mineral oil, and cultured for 44 h under the same conditions. Oocyte maturation rate was calculated as the percentage of metaphase II (MII) oocytes relative to total cultured oocytes.

Metformin hydrochloride was dissolved in sterile ultrapure water to prepare a 1 M stock solution. The Nrf2-specific inhibitor ML385 (SML1833, Aladdin, Shanghai, China) was dissolved in dimethyl sulfoxide (DMSO) to prepare a 5 mM stock solution. Both stock solutions were aliquoted and stored at −20 °C to avoid repeated freeze–thaw cycles. Metformin and ML385 were added directly into IVM medium at the beginning of in vitro maturation culture. In all treatment groups, drugs or vehicle were supplemented at 0.1% (*v*/*v*) of the total medium volume. Control groups were treated with 0.1% (*v*/*v*) DMSO (the solvent used to dissolve ML385), a concentration previously verified to have no significant effects on porcine oocyte maturation and subsequent embryonic development in our prior work [23].

Following maturation culture, oocytes were vortexed in 1 mg/mL hyaluronidase for 3 min at 37 °C to remove cumulus cells, and the resulting denuded oocytes (DOs) were collected for subsequent experiments. The number of oocytes that released the first polar body was counted to calculate the oocyte maturation rate, which was then used to evaluate the maturation quality and developmental competence of the oocytes. At least six independent biological replicates were included to ensure statistical validity. (*n* = 163–194 COCs). Each MET concentration treatment was repeated 6–8 times with approximately 25 oocytes per replicate. The total number of oocytes used in each group was 157 (0 μM), 167 (7.5 μM), 194 (15 μM), 172 (30 μM), 206 (150 μM), and 160 (300 μM), respectively.

### 2.2. Oocyte Parthenogenetic Activation and Embryo Development

Mature denuded oocytes were first treated with 5 µM ionomycin contained in D-PBS for 5 min in the dark. Then, the oocytes were washed three times in the porcine zygote medium-3 (PZM-3) medium and incubated for 5 h in PZM-3 medium containing 2 mM 6-DMAP (12–16 oocytes per drop). At the end of the incubation, 6-DMAP was removed by washing the oocytes in D-PBS and the activated oocytes were transferred into 150 mL mineral-oil-covered PZM-3 (12–16 oocytes per drop) and cultured at 38.5 °C under 5% CO_2_ in humidified air for 7 days. Cleavage, 4-cell, and blastocyst rates were evaluated at 24 h, 48 h, and 168 h post-parthenogenetic activation (PA), respectively. Cleavage rate (2-cell stage) was the percentage of 2-cell embryos relative to total matured oocytes. The 4-cell embryo rate was the percentage of 4-cell embryos relative to total cleaved embryos. Blastocyst rate was the percentage of blastocysts relative to total 4-cell embryos.

### 2.3. Assessment of Cortical Granule Distribution

The zona pellucida of oocytes was removed using acidic Tyrode’s solution (GL2164, Biolab Biotechnology Co., Ltd., Beijing, China). Oocytes were washed three times with D-PBS, fixed in 4% paraformaldehyde (PFA) for 30 min at room temperature, and then subjected to blocking. For blocking, oocytes were incubated three times (5 min each) in blocking solution (D-PBS supplemented with 0.3% BSA and 100 mM glycine). For permeabilization, oocytes were treated with D-PBS containing 0.1% Triton X-100 for 5 min, followed by washing twice with blocking solution (5 min each). For fluorescent staining, oocytes were incubated with 100 μg/mL FITC-LCA (FL-1041-5, Vector Laboratories, Burlingame, CA, USA) in D-PBS for 30 min in the dark, then washed three times with D-PBS (5 min each). Afterward, oocytes were counterstained with Hoechst 33342 for nuclear labeling, mounted in anti-fade mounting medium, and covered with a coverslip. Images were captured using a confocal microscope, and the fluorescence intensity of cortical granules was quantified with excitation at 488 nm and emission at 500–550 nm for the FITC (CG) channel, and excitation at 405 nm and emission at 430–480 nm for the Hoechst 33342 (nuclear) channel. Cortical granule (CG) distribution patterns were classified as normal or abnormal: normal distribution was defined as continuous, dense CG localization beneath the oolemma with minimal signal in the inner cytoplasm; abnormal distribution was characterized by discontinuous or incomplete CG labeling at the plasma membrane, accompanied by diffuse CG signal remaining within the ooplasm. The proportion of oocytes with abnormal CG distribution was calculated accordingly. Each treatment was repeated 5 times with approximately 20 oocytes per replicate. The total number of oocytes observed for cortical granule distribution was 88 (0 μM), 91 (15 μM), and 94 (300 μM), respectively.

### 2.4. One-Step RT qPCR for Oocyte Gene Expression Analysis

Measurement of gene expression with quantitative real-time PCR has been described [24]. Total RNA was isolated from 60 DOs using a direct lysis strategy. Following washing, the oocytes were lysed in 30 μL of FlysisAmp Lysis Buffer (CL132, Vazyme, Nanjing, China) supplemented with DNase I. After termination of the lysis reaction, the lysate was stored at −80 °C. One step RT qPCR amplification was conducted in a 10 μL system using the Mx3005P system (Stratagene, Valencia, CA, USA). The reaction conditions consisted of reverse transcription at 55 °C for 3 min, pre denaturation at 95 °C for 3 s, and 40 cycles of amplification (95 °C for 5 s and 60 °C for 20 s). Gene specific primers are listed in Table 1, and relative expression levels were analyzed using the 2^−ΔΔCT^ method, with β-actin as the reference gene for normalization. For all qRT-PCR analyses, 60 DOs were collected per sample, with 3 independent biological replicates per treatment group. The same cDNA sample synthesized from one biological replicate was used for quantitative real time PCR analysis of all 7 target genes and 1 reference gene. A total of 720 DOs were used for all gene expression assays in this study.

### 2.5. Untargeted Metabolomics Sequencing Based on LC–MS

For untargeted metabolomics analysis, 400 denuded oocytes were collected per biological replicate after 44 h of IVM. Three independent biological replicates were prepared for each group (control and 15 μM MET). In total, 1200 DOs were harvested for each of the two experimental groups, leading to a total of 2400 DOs across the entire metabolomics experiment. For metabolomics sample preparation, collected denuded oocytes were washed with ice-cold PBS, centrifuged at 1000× *g* at 4 °C for 5 min, and washed three additional times with ice-cold PBS. Pellets were snap-frozen in liquid nitrogen for 1 min and stored at −80 °C. For protein precipitation and metabolite extraction, samples were processed with 1 mL ice-cold methanol/acetonitrile/water (2:2:1, *v*/*v*), sonicated in an ice bath for 1 h, incubated at −20 °C for 1 h, then centrifuged at 14,000× *g* at 4 °C for 20 min. The extracted metabolites were pre separated by liquid chromatography (LC) and subsequently detected and analyzed by mass spectrometry (MS) at Shanghai Bioprofile Technology Co., Ltd. (Shanghai, China) (http://www.bioprofile.cn/; accessed on 18 December 2025). The detection intensity of each metabolite across the two groups was obtained and subjected to omics analysis using the Metware Cloud platform (https://cloud.metware.cn; accessed on 20 December 2025). Differential metabolites between the control and 15 μM metformin treated groups were identified using the criteria of variable importance in projection (VIP) > 1 and *p* < 0.1. Heat maps and KEGG pathway enrichment bubble plots were generated and analyzed using the online tools provided by the platform. The complete list of differential metabolites is provided in Table S1.

### 2.6. Detection of ROS

Intra-oocyte reactive oxygen species (ROS) levels were measured using 10 μM 2′,7′-dichlorodihydrofluorescein diacetate (DCFH-DA, HY-D0940, MCE, Monmouth Junction, NJ, USA). Oocytes were incubated with the probe at 37 °C for 10 min in the dark, washed three times with D-PBS, and immediately observed and imaged using a Leica fluorescence microscope. Fluorescence signals were detected at an excitation wavelength of 488 nm, and all images were captured with fixed microscope parameters to ensure data consistency. Fluorescence intensity of individual oocytes was analyzed using ImageJ software (version 1.53t, National Institutes of Health, Bethesda, MD, USA). Each treatment was repeated 4 times with approximately 25 oocytes per replicate. The total number of oocytes detected for intracellular ROS levels was 97 (0 μM), 89 (15 μM), and 91 (300 μM), respectively.

### 2.7. Detection of MMP

Mitochondrial membrane potential in porcine oocytes was measured using the JC-1 (JC-1 cationic fluorescent probe) MMP detection kit (C2006, Beyotime Biotechnology, Shanghai, China). Briefly, oocytes matured in vitro for 44 h were denuded of cumulus cells by hyaluronidase treatment, and oocytes with uniform cytoplasm were selected and washed three times with PBS to remove residual debris. Oocytes were then incubated in pre-warmed JC-1 working solution for 30 min in the dark, followed by three washes with JC-1 staining buffer.

Images were captured using a Leica laser scanning confocal microscope. JC 1 aggregates (red fluorescence) were detected at 590 nm using the TRITC channel, while JC 1 monomers (green fluorescence) were detected at 512 nm using the FITC channel. All imaging parameters were standardized across all experimental groups to ensure consistent data acquisition. The red to green fluorescence intensity ratio was quantified using ImageJ software, with approximately 25 mature oocytes analyzed per group and four independent biological replicates performed. The total number of oocytes detected for mitochondrial membrane potential was 94 (0 μM), 98 (15 μM), and 86 (300 μM), respectively.

### 2.8. Detection of AMP and ATP Content in Oocytes

Intracellular levels of adenosine monophosphate (AMP) and adenosine triphosphate (ATP) were measured using porcine AMP ELISA kit (JB492-Pg) and porcine ATP ELISA kit (JB455-Pg) (Jinma Biotech, Shanghai, China), following the manufacturer’s instructions.

Briefly, 50 porcine oocytes were collected per group and suspended in 100 μL of pre cooled PBS. Oocytes were lysed by three cycles of repeated freeze thawing, then centrifuged at 10,000× *g* for 10 min at 4 °C. The supernatant was collected as the test sample, diluted with the provided sample diluent, and added to microplate wells.

After incubation at 37 °C, washing, enzyme labeling, and color development in the dark, the reaction was terminated, and the optical density (OD) was measured at 450 nm using a microplate reader. The concentrations of AMP and ATP were calculated against standard curves, and the AMP/ATP ratio was used to evaluate cellular energy status. All experiments were performed in three independent biological replicates. Each replicate included 50 DOs per group (3 groups total), resulting in 450 DOs in total.

### 2.9. Western Blot

For protein extraction, 100 denuded oocytes were placed in a centrifuge tube and lysed in 20 μL of lysis buffer containing 95 μL of 2× Laemmli Sample Buffer (1610737, Bio Rad, Hercules, CA, USA), 5 μL of 2-mercaptoethanol, 1 μL of protease inhibitor cocktail (HY-K0010, MCE, Monmouth Junction, NJ, USA), and 1 μL of phosphatase inhibitor cocktail (CW2383S, Cwbio, Taizhou, China). Lysis was performed on ice for 30 min at 4 °C, with gentle vortexing every 10 min. After lysis, samples were denatured at 95 °C for 5 min using a dry bath heater.

The denatured proteins were subjected to SDS-PAGE gel separation of proteins and transferred to PVDF membranes, which were then blocked with 5% skimmed milk for 90 min. Next, the addition of primary antibody was added and incubated overnight at 4 °C. The primary antibodies used were from ABclonal Biotechnology (Wuhan, China), including AMPK (A12718SP), pAMPK (AP0432), Nrf2 (A0674SP) (1:1000 dilution), and GAPDH (A19056) (1:10,000 dilution). The membranes were subsequently incubated for 50 min at room temperature in a secondary antibody containing horseradish peroxidase (HRP) labeled anti-rabbit IgG. The ECL reagent (Cheml Scope5300, Clinx Science Instruments, Shanghai, China) was added to the membrane, and the fluorescent signals were detected by the image VCD gel imaging system (Clinx Science Instruments, Shanghai, China). For Western blotting analysis, 100 denuded oocytes were collected per sample, with 3 independent biological replicates per treatment group. A total of 900 DOs were used for all Western blotting assays in this study.

### 2.10. Quantification and Statistical Analysis

Data were analyzed using SPSS 27.0 (SPSS Inc., Chicago, IL, USA) and GraphPad Prism software (version 6.0, GraphPad Software, San Diego, CA, USA). Comparisons among three or more groups were performed using one way ANOVA followed by Duncan’s multiple range test, while comparisons between two groups were analyzed using the independent samples *t* test. All data are expressed as means ± standard error of the mean (SEM). Differences were considered statistically significant at *p* < 0.05.

### 2.11. Experimental Design

Each experiment was independently replicated at least three times unless otherwise stated. This study explored the concentration-dependent biphasic effects and molecular mechanisms of MET on porcine oocyte IVM. All experiments were performed using independent biological replicates, and no oocyte samples were shared between different phenotypic or molecular assays. The same cDNA sample synthesized from one biological replicate could be used for quantitative real-time PCR analysis of multiple target genes. The overall experimental workflow was divided into five sequential parts as follows.

#### 2.11.1. Experiment 1: Screening of Effective MET Concentrations

To screen effective and detrimental MET concentrations for oocyte IVM, COCs were randomly exposed to six gradient MET treatments (0, 7.5, 15, 30, 150, and 300 μM). After 44 h of IVM, oocyte maturation, cleavage, and blastocyst formation rates were statistically analyzed. According to the phenotypic outcomes, 15 μM MET and 300 μM MET were determined as the optimal low-dose and detrimental high-dose concentrations, respectively, for all subsequent mechanistic experiments.

#### 2.11.2. Experiment 2: Evaluation of Oocyte Cytoplasmic Maturation

The effects of low and high MET doses on oocyte cytoplasmic maturation and developmental competence were evaluated using three treatment groups (control, 15 μM MET, 300 μM MET). After IVM culture, CG distribution was observed to assess cytoplasmic maturation status, and qRT-PCR was performed to detect the transcript levels of oocyte developmental competence-related genes, including *Mater*, *Zar1*, and *GDF9*.

#### 2.11.3. Experiment 3: Untargeted Metabolomics Profiling to Identify Key Regulatory Pathways Mediating the Beneficial Effects of Low-Dose MET

Untargeted LC–MS-based metabolomics was conducted using control and 15 μM MET-treated oocytes to investigate metabolic alterations induced by low-dose MET. Differentially expressed metabolites and enriched pathways were screened, with a focus on energy metabolism and antioxidant defense pathways. The metabolomic findings provided core clues and directed the subsequent validation of the AMPK–Nrf2 signaling axis.

#### 2.11.4. Experiment 4: Assessment of Redox Homeostasis

Guided by the metabolomic pathway characteristics, the regulatory effects of MET on oocyte redox balance and mitochondrial function were further verified in control, 15 μM MET, and 300 μM MET groups. We detected intracellular ROS levels and mitochondrial membrane potential to evaluate redox status and quantified the expression of antioxidant-related genes (GPX4, CAT, SOD1, Nrf2) via qRT-PCR.

#### 2.11.5. Experiment 5: AMPK–Nrf2 Signaling Pathway Validation and Functional Rescue

Based on the above phenotypic and molecular results, we further validated the activation and function of the AMPK–Nrf2 pathway. The cellular energy status of oocytes from the three MET treatment groups was assessed by detecting AMP and ATP contents and calculating the AMP/ATP ratio. Western blotting was used to determine the protein levels of Nrf2, p-AMPK, and total AMPK. Furthermore, an Nrf2 inhibitor (ML385) rescue assay with four groups (control, 15 μM MET, ML385, 15 μM MET + ML385) was performed to confirm the functional role of Nrf2 in mediating MET-regulated oocyte maturation and parthenogenetic embryo development.

## 3. Results

### 3.1. Dose-Dependent Effects of Metformin on Porcine Oocyte In Vitro Maturation and Developmental Competence

**Figure 1:**
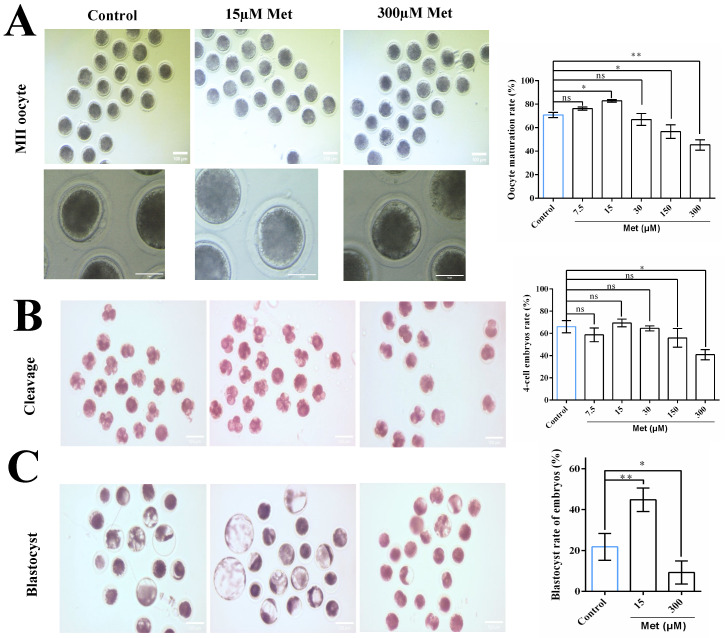
Effects of metformin on oocyte maturation and subsequent embryo development. (**A**) Representative images of oocytes matured for 44 h in control, 15 µM metformin (Met), or 300 µM Met conditions, and quantification of oocyte maturation rate. (**B**) Representative images of embryo cleavage and quantification of cleavage rate after insemination of matured oocytes (conditions as in (**A**)). (**C**) Representative images of blastocyst formation and quantification of blastocyst rate in embryos from each treatment group (conditions as in (**A**)). Percentages of oocyte maturation, 4-cell embryos, and blastocysts were calculated from cultured oocytes, 2-cell embryos, and 4-cell embryos, respectively. Data are presented as the mean ± SEM of at least three independent experiments. * *p* < 0.05, ** *p* < 0.01, ns (not significant) indicates no statistically significant difference.

As shown in Figure 1A, the percentage of MII oocytes was significantly increased in the 15 μM Met group (82.85 ± 1.05%, *p* = 0.0413) compared with the control group (70.80 ± 2.20%), while higher concentrations (≥150 μM) led to a dose-dependent reduction in maturation rate (e.g., 56.68 ± 5.71% at 150 μM, *p* = 0.0337; 45.34 ± 4.38% at 300 μM, *p* = 0.0081). Following PA, the blastocyst formation rate was remarkably elevated in the 15 μM Met group (44.83 ± 5.76%) relative to the control group (23.81 ± 6.54%, *p* = 0.0091; Figure 1C), while no significant differences were observed in the cleavage rate (Figure 1B) or 4 cell/2 cell ratio between groups. In contrast, concentrations ≥ 150 μM severely compromised blastocyst formation (e.g., 9.26 ± 5.61% at 300 μM, *p* = 0.0327 vs. control). Accordingly, based on comparisons with the control group, 15 μM metformin was defined as the low concentration and 300 μM as the high concentration for subsequent experiments.

**Figure 2:**
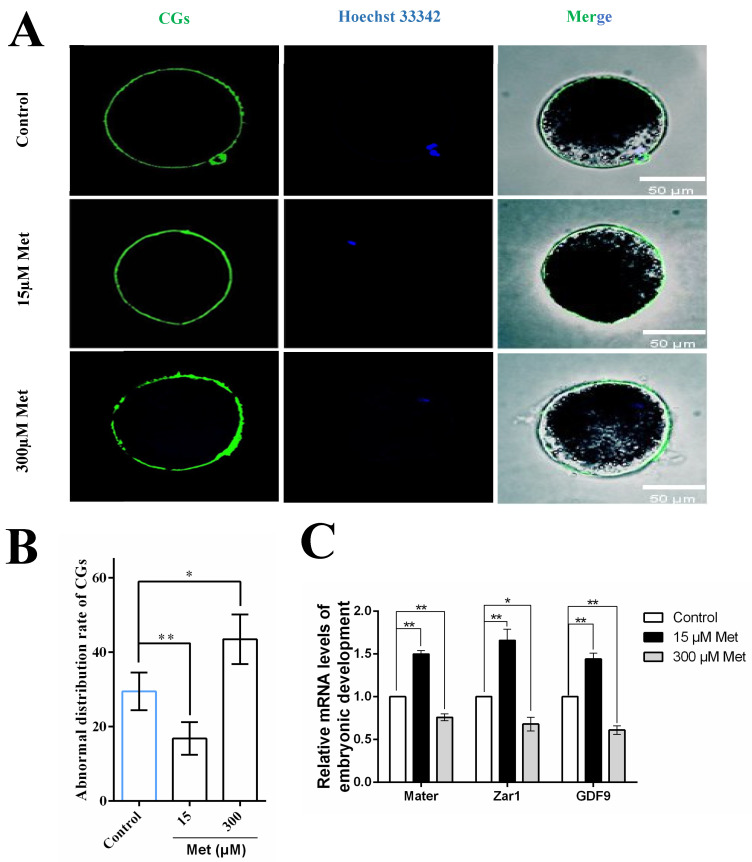
Effects of metformin on cortical granule distribution and oocyte developmental competence-related gene expression. (**A**) Fluorescence images of oocytes (from each group) stained for cortical granules (CGs; green), Hoechst 33342 (nuclear staining; blue), and merged channels (scale bar = 50 µm). (**B**) The proportion of oocytes exhibiting abnormal CG localization after 44 h of culture in control, 15 µM Met, or 300 µM Met groups. (**C**) Relative mRNA levels of oocyte development-related genes. Data are presented as the mean ± SEM of at least three independent experiments. * *p* < 0.05, ** *p* < 0.01.

As shown in Figure 2B, the proportion of oocytes with abnormal CG distribution was significantly reduced in the 15 μM Met group (18.78 ± 0.02%) compared with the control group (27.38 ± 0.01%, *p* = 0.0082), while the 300 μM Met group had a higher rate of abnormal CG distribution (46.24 ± 0.02%, *p* = 0.0331 vs. control). To further assess oocyte developmental competence, we analyzed the expression of three key genes associated with oocyte quality: *Mater*, *Zar1*, and *GDF9*. As illustrated in Figure 2C, compared with the control group (normalized to 1.00), the 15 μM Met group exhibited significantly increased mRNA levels of *Mater*, *Zar1* and *GDF9* (*p* < 0.01), while these genes were markedly downregulated in the 300 μM Met group (*p* < 0.05).

### 3.2. Low-Concentration Metformin Remodels Metabolic Homeostasis in Porcine Oocytes

**Figure 3:**
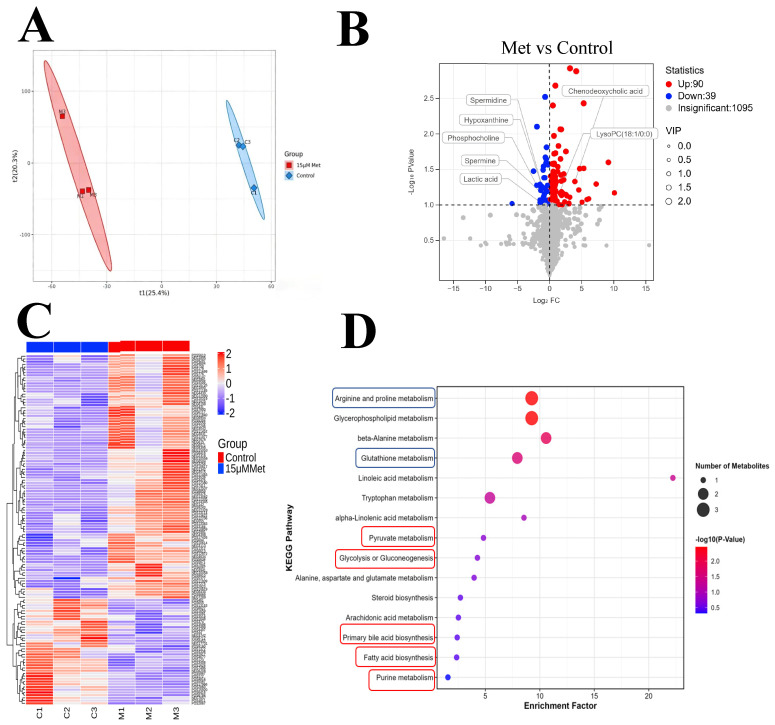
Low-concentration metformin reprograms the metabolic profile of porcine oocytes during in vitro maturation. (**A**) PLS-DA score plot of control and 15 μM metformin (Met) groups. (**B**) Volcano plot of differential metabolites (VIP > 1, *p* < 0.1). Blue: upregulated (90); red: downregulated (39). (**C**) Heatmap of the 129 differential metabolites. (**D**) KEGG enrichment analysis of the top 15 enriched pathways. Red solid boxes: AMPK-associated pathways. Blue solid boxes: antioxidant stress pathways.

Differential metabolites between the control and Met (15 μM) groups were identified using partial least squares-discriminant analysis (PLS-DA), from which variable importance in projection (VIP) values were derived. Compounds with VIP > 1 were considered potential biomarkers contributing to group separation (Figure 3A). A total of 129 differential metabolites were identified (VIP > 1, *p* < 0.1), with 90 upregulated and 39 downregulated in the metformin group compared to the control group (Figure 3B). KEGG pathway enrichment analysis of these differential metabolites (between the metformin and control groups) revealed pathways: those functionally associated with AMPK signaling, including fatty acid biosynthesis, purine metabolism, primary bile acid biosynthesis, and glycolysis/gluconeogenesis; and those representing canonical downstream targets of antioxidant defense pathways, including arginine and proline metabolism and glutathione metabolism (Figure 3D). Consistent with improved oocyte quality, metabolites favoring AMPK activation and antioxidant defense were significantly modulated. Notably, the AMPK activator chenodeoxycholic acid was strikingly elevated (fold change, FC = 15.23, *p* = 0.0468), accompanied by increased LysoPC (18:1/0:0) (FC = 1.89, *p* = 0.0921), an oxidized lipid known to induce Nrf2 responses. Spermidine (FC = 0.45, *p* = 0.0552) and spermine (FC = 0.55, *p* = 0.0733) were dynamically altered, likely reflecting their active engagement in cytoprotective processes. Additional changes included hypoxanthine (FC = 0.57, *p* = 0.0698), lactic acid (FC = 0.50, *p* = 0.0982), and phosphocholine (FC = 0.25, *p* = 0.0528) (Figure 3B). Hierarchical clustering confirmed distinct metabolite profiles between groups (Figure 3C).

### 3.3. Metformin Regulates Redox Balance and Mitochondrial Homeostasis in a Concentration-Dependent Manner During Porcine Oocyte Maturation

**Figure 4:**
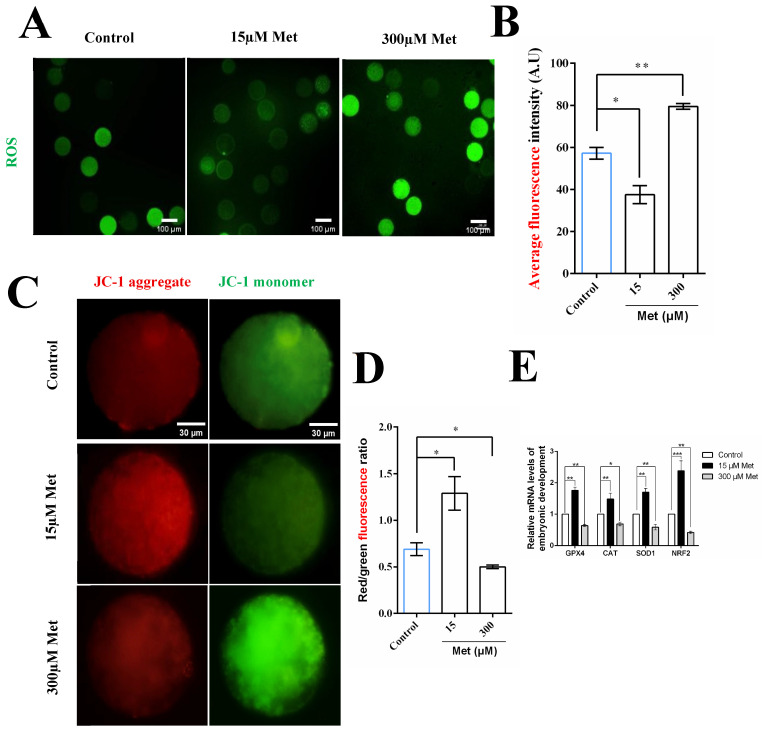
Effects of metformin on redox potential in porcine oocytes. (**A**) Representative images of oocytes matured for 44 h in control, 15 μM metformin (Met), or 300 μM Met conditions, stained to detect reactive oxygen species (ROS). Scale bars are indicated in the images. (**B**) Quantitative analysis of ROS fluorescence intensity in oocytes from the three groups. (**C**) Representative JC-1 staining images (red: high mitochondrial membrane potential; green: low mitochondrial membrane potential) of oocytes in each group. Scale bars are indicated in the images. (**D**) Quantitative analysis of JC-1 red/green fluorescence ratio (reflecting mitochondrial membrane potential) in oocytes from the three groups. (**E**) Relative mRNA levels of redox-related genes (*GPX4*, *CAT*, *SOD1*, *Nrf2*) in oocytes from the three groups. Data are presented as the mean ± SEM of at least three independent experiments. * *p* < 0.05, ** *p* < 0.01, *** *p* < 0.001.

To explore the effects of Met on redox balance and mitochondrial function in porcine oocytes, we first measured intracellular reactive oxygen species (ROS) levels. As shown in Figure 4B, the ROS level (average fluorescence intensity, AFIV) was significantly lower in the 15 μM Met group (37.54 ± 1.27) compared with the control group (57.22 ± 2.78, *p* = 0.0258), while the 300 μM Met group exhibited a markedly higher ROS level (79.50 ± 1.38, *p* = 0.0079 vs. control).

Next, we assessed mitochondrial membrane potential (ΔΨm) using JC 1 staining. As illustrated in Figure 4D, the ratio of red to green fluorescence (a marker of ΔΨm) was significantly increased in the 15 μM Met group (1.29 ± 0.18, *p* = 0.0267) relative to the control group (0.69 ± 0.07), indicating enhanced mitochondrial function. In contrast, the 300 μM Met group showed a significantly reduced ratio (0.50 ± 0.20, *p* = 0.0458), suggesting mitochondrial dysfunction.

Consistent with these findings, the expression of antioxidant enzyme genes was analyzed by qRT PCR. As presented in Figure 4E, compared with the control group (set to 1.00), the 15 μM Met group displayed upregulated mRNA levels of *GPX4* (1.76 ± 0.10), *CAT* (1.48 ± 0.19), *SOD1* (1.70 ± 0.12), and *Nrf2* (2.38 ± 0.32) (*p* < 0.01 for all). Conversely, the 300 μM Met group showed decreased expression of all these genes (*GPX4*: 0.64 ± 0.03; *CAT*: 0.69 ± 0.06; *SOD1*: 0.59 ± 0.09; *Nrf2*: 0.42 ± 0.04) (*p* < 0.05 for all). These results demonstrate that low-concentration (15 μM) metformin alleviates oxidative stress, preserves mitochondrial function, and upregulates antioxidant gene expression in porcine oocytes, whereas high-concentration (300 μM) metformin exacerbates oxidative damage and impairs mitochondrial homeostasis.

### 3.4. Low-Concentration Metformin Activates AMPK Independently of AMP/ATP Ratio via the AMPK–Nrf2 Axis

**Figure 5:**
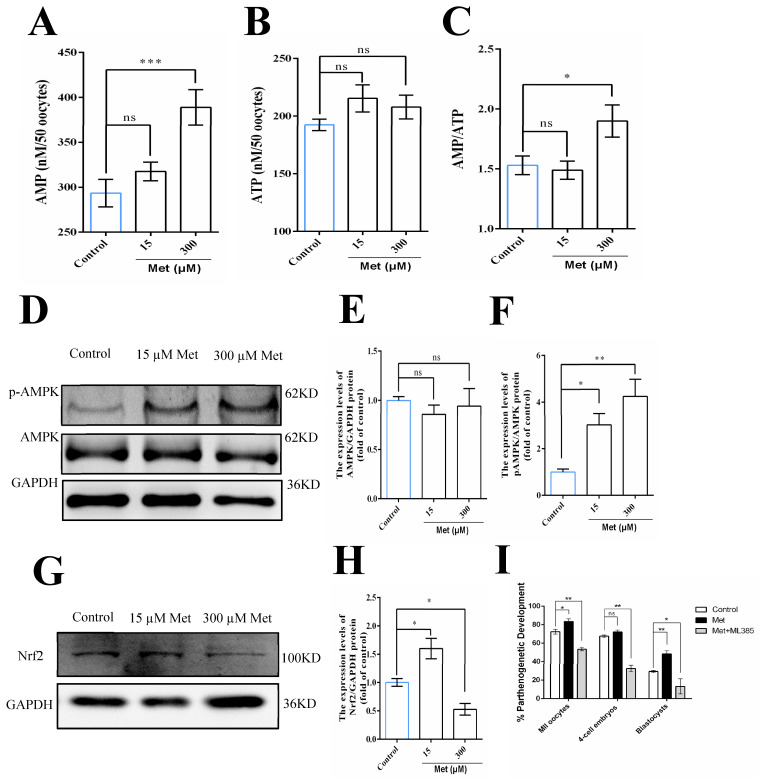
Low-concentration metformin activates AMPK via an AMP/ATP ratio-independent pathway and improves porcine oocyte maturation and developmental competence through the AMPK–Nrf2 axis, validated by Nrf2 inhibitor ML385 (5 µM). (**A**–**C**) Quantitative analysis of intracellular AMP content (**A**), ATP content (**B**), and AMP/ATP ratio (**C**) in porcine oocytes from control, low-concentration Met (15 μM), and high-concentration Met (300 μM) groups, detected by commercial AMP and ATP assay kits. (**D**,**G**) The expression levels of p-AMPK, total AMPK, Nrf2 and GAPDH in porcine oocytes treated with 0 (Control), 15 μM, or 300 μM metformin. (**E**,**F**,**H**) Relative quantification of Western blots shown in (**D**,**G**). (**I**) Effects of Met and the Nrf2 inhibitor ML385 (5 μM) on parthenogenetic development of porcine oocytes. Data are presented as the mean ± SEM of at least three independent experiments. * *p* < 0.05, ** *p* < 0.01, *** *p* < 0.001, ns (not significant) indicates no statistically significant difference.

First, we assessed whether Met activates AMPK via the canonical AMP/ATP ratio-dependent pathway. As shown in Figure 5A–C, 15 μM Met did not significantly alter intracellular AMP or ATP content, nor the AMP/ATP ratio (all *p* > 0.05), while 300 μM Met significantly elevated AMP levels and the AMP/ATP ratio without changing ATP levels. These results confirm that low-concentration Met activates AMPK through an AMP/ATP ratio-independent mechanism.

Next, we validated AMPK–Nrf2 pathway activation via Western blotting (representative blots in Figure 5D,G). Quantitative analysis revealed that total AMPK protein expression was unchanged across all groups (Figure 5E), but phosphorylated AMPK (p-AMPK, the active form) was significantly upregulated in both Met-treated groups: 15 μM Met increased p-AMPK levels (*p* = 0.0318 vs. control), and 300 μM Met induced a more pronounced elevation (*p* = 0.0043 vs. control) (Figure 5F). Nrf2 protein expression, a master regulator of antioxidant defense, was significantly upregulated in the 15 μM Met group (*p* = 0.0154 vs. control) but downregulated in the 300 μM Met group, compared with the control (*p* = 0.0379) (Figure 5H).

To functionally confirm that Nrf2 mediates the beneficial effects of low-concentration Met on oocyte quality, we performed rescue experiments with the specific Nrf2 inhibitor ML385 (5 µM). As shown in Figure 5I, 15 μM Met significantly improved the rates of MII oocytes and blastocysts compared with the control, mirroring its protective effects against oxidative stress. Critically, co-treatment with ML385 completely abrogated these benefits: the Met + ML385 group exhibited significantly lower developmental rates at all stages relative to the Met-only group, with levels returning to baseline or below control values. Together, 15 μM Met does not affect AMP, ATP content, or AMP/ATP ratio, but upregulates p-AMPK and Nrf2; Nrf2 inhibitor abolishes its beneficial effects, and300 μM Met increases AMP and AMP/ATP ratio and impairs oocyte quality.

## 4. Discussion

**Figure 6:**
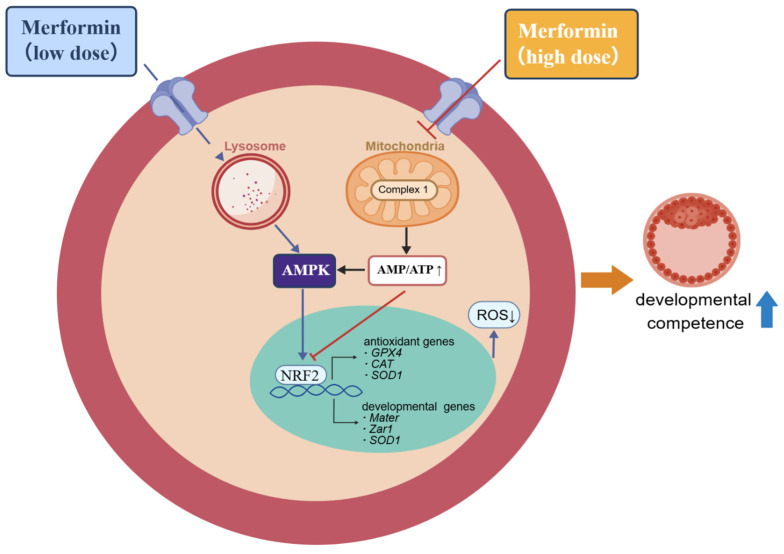
Schematic diagram illustrating the concentration-dependent biphasic effects of metformin on porcine oocyte developmental competence.

Despite the well-documented regulatory roles of metformin (Met) in oocyte meiosis, previous studies have primarily focused on the inhibitory effects of high-concentration Met on meiotic progression, such as germinal vesicle breakdown (GVBD) arrest and meiotic resumption block [13,14,15,25], with limited attention paid to its concentration-dependent effects on oocyte developmental competence. Our findings demonstrated that low-dose metformin (15 μM) significantly improved porcine oocyte nuclear and cytoplasmic maturation, whereas high-dose metformin (300 μM) exerted detrimental effects. These biphasic effects are consistent with previous reports in mice and pigs. In mice, Cao et al. [26] showed that 10–50 μM metformin improved oocyte maturation and mitochondrial function, while Sun et al. [14] found that concentrations above 2000 μM induced oxidative stress and spindle damage. Similarly, in pigs, Zhou et al. [21] reported that 400 μM metformin protected oocytes against cryoinjury, whereas Bilodeau-Goeseels et al. [15] demonstrated that 2000 μM metformin inhibited germinal vesicle breakdown. Prior studies have focused primarily on metformin’s effects during meiotic progression, with limited data addressing its dose-dependent impacts on subsequent embryonic development. Collectively, our findings confirm that metformin regulates the quality of porcine oocytes in a concentration-dependent manner (Figure 6).

Notably, our mechanistic data revealed that low-dose metformin exerted beneficial effects by targeting the lysosomal PEN2–v–ATPase complex to activate AMPK independently of cellular energy stress, without altering AMP, ATP, or the AMP/ATP ratio [20]. This noncanonical pathway is consistent with recent landmark studies demonstrating that low, clinically relevant concentrations of metformin extend lifespan in C. elegans [27] and mice [28] by modulating lysosomal function and energy metabolism without inducing overt energy stress. In contrast, high-dose metformin (300 μM) directly inhibited mitochondrial respiratory chain complex I, impaired oxidative phosphorylation, elevated the AMP/ATP ratio, and triggered energy-stress-dependent AMPK activation [19,29], which was associated with excessive ROS accumulation, oxidative damage, mitochondrial dysfunction, and spindle impairment [30,31]. Consistent with Feng et al. [32], excessive metformin potently suppresses mitochondrial complex I to interrupt respiratory chain transmission, driving massive ROS overproduction and energy imbalance. Kamble et al. [33] further confirmed that such mitochondrial perturbation triggers sustained pathological AMPK overactivation and redox collapse. We demonstrated that supplementation with low-dose MET during in vitro maturation of porcine oocytes activates AMPK through a noncanonical pathway independent of changes in the AMP/ATP ratio, thereby improving oocyte maturation and embryonic developmental competence.

Interestingly, inconsistent effects of low-concentration metformin have been reported in porcine oocyte IVM systems. Lee et al. [22] found 10 μM metformin showed no standalone beneficial effect but enhanced insulin-mediated improvements in blastocyst formation and GSH content, whereas 15 μM metformin alone significantly promoted oocyte maturation and embryonic development in our study. This discrepancy is mainly driven by differences in basal IVM medium composition: Lee’s culture medium was supplemented with porcine follicular fluid, serum, and endogenous insulin-like factors that offset independent metformin function, while our defined IVM system with reduced follicular fluid allowed metformin to trigger AMPK–Nrf2 signaling and independently optimize oocyte redox balance and developmental potential.

Untargeted metabolomic profiling identified differential metabolites that were significantly enriched in pathways closely associated with AMPK signaling and Nrf2-mediated antioxidant responses, including purine metabolism, glutathione metabolism, and oxidative phosphorylation (Figure 3D). These metabolic pathways are tightly regulated by AMPK activity and are essential for Nrf2-driven redox homeostasis. Collectively, these metabolomic data indicate that low-dose metformin exerts its beneficial effects primarily by modulating the AMPK–Nrf2 signaling axis, which is consistent with reports that AMPK acts as an upstream activator to promote Nrf2 nuclear translocation and antioxidant activity via phosphorylation of GSK-3β [34]. Published porcine germ cell data reveal that functional AMPK phosphorylates Nrf2 to disrupt the Keap1–Nrf2 complex. Free Nrf2 translocates into the nucleus and binds ARE motifs to induce antioxidant gene transcription [35]. We further verified this pathway using the specific Nrf2 inhibitor ML385. As shown in Figure 5H, the Met + ML385 group exhibited significantly lower oocyte maturation and blastocyst formation rates than the control group, likely due to the dual inhibitory effect of ML385: it not only blocked the upregulation of Nrf2 induced by low-dose metformin but also suppressed endogenous basal Nrf2 activity, which is essential for maintaining physiological redox balance during IVM [36,37].

## 5. Conclusions

This exploratory study demonstrates a concentration-dependent biphasic effect of metformin on porcine oocyte in vitro maturation and subsequent embryonic development. Low-dose metformin (15 μM) improves oocyte quality by triggering non-canonical AMPK activation independent of energy stress, further activating the AMPK–Nrf2 antioxidant signaling axis to relieve oxidative damage. In contrast, high-dose metformin (300 μM) induces cellular energy stress and exerts adverse impacts on oocyte maturation and developmental capacity. Our findings define the optimal metformin concentration for porcine oocyte IVM and reveal that the AMPK–Nrf2 pathway contributes to the beneficial effects of low-dose metformin on oocyte developmental competence. These results provide a theoretical basis and practical strategy for optimizing in vitro culture systems and improving the efficiency of in vitro embryo production in pigs.

## Figures and Tables

**Table 1:** Oligonucleotide primer sequences used for real-time PCR in this study.

cDNA	Gene ID	Oligonucleotide Sequences (5′–3′)	Amplified Product Size (bp)
*β-actin*	414396	F: CGTGCGGGACATCAAGGA	177
		R: AGGAAGGAGGGCTGGAAGA	
*GDF9*	414285	F: GATTGATGTGACGGCCATCC	183
		R: TGTGTGCTTGTGTCGTTCAG	
*Mater*	100170773	F: AGCTGATACTCGGGGACTGT	229
		R: GCAAATGCAAGGAAGCCACA	
*Zar1*	574050	F: TGGTGTGTCCAGGGCACTAA	213
		R: GTCACAGGAGAGGCGTTTGC	
*Bcl2*	100049703	F: GACTTCTCTCGTCGCTACCG	283
		R: CATCCCAGCCTCCGTTATCC	
*Bax*	396633	F: AGTGGCGGCCGAAATGTTTG	166
		R: CAGCAGCCGATCTCGAAGGA	
*CAT*	397568	F: ACATGGTCTGGGACTTCTGG	99
		R: TCATGTGCCTGTGTCCATCT	
*GPX4*	399537	F: ATTCTCAGCCAAGGACATCG	93
		R: CCTCATTGAGAGGCCACATT	
*NRF2*	100516343	F: CATAGCAGAGCCCAGTACCA	134
		R: CACGGTGGTCTTGGTTGAAG	
*SOD1*	397036	F: TCCATGTCCATCAGTTTGGA	131
		R: AGTCACATTGCCCAGGTCTC	

## Data Availability

The original contributions presented in this study are included in the article/Supplementary Materials. Further inquiries can be directed to the corresponding author.

## Supplementary Materials

The following supporting information can be downloaded at: https://www.mdpi.com/article/10.3390/ani16121828/s1, Table S1: Differential metabolites; Table S2: Effects of metformin on porcine oocyte maturation and early embryonic development.

## Abbreviations

The following abbreviations are used in this manuscript:

AMPK	AMP-activated protein kinase
PCOS	Polycystic ovary syndrome
MET	Metformin
MII	Metaphase II
IVM	In vitro maturation
IVP	In vitro production
IVF	In vitro fertilization
IVC	In vitro culture
ROS	Reactive oxygen species
GPD2	Glycerophosphate dehydrogenase
COCs	Cumulus–oocyte complexes
CG	Cortical granule
DOs	Denuded oocytes
PA	Parthenogenetic activation
PLS-DA	Partial least squares-discriminant analysis
FC	Fold change
