# Supplementary material for: Biphasic Responses of Porcine Oocytes to Metformin: Concentration-Dependent AMPK Activation and Nrf2-Mediated Antioxidant Regulation
Source: Animals (Basel). 2026 Jun 13;16(12):1828. doi: 10.3390/ani16121828 (PMC13295701; doi:10.3390/ani16121828)
Supplement: Supplementary file 1 [file animals-16-01828-s001.zip › animals-4330428-Table S2.pdf]

Supplementary Table 2

Effects of metformin on porcine oocyte maturation and early embryonic development.

MET （μM）	No. COC	MⅡ oocyte rate （% ± SEM）	Cleavage rate （% ± SEM）	4-cell embryo rate （% ± SEM）	Blastocyst rate （% ± SEM）
0	157	70.80±2.20[a]	73.89±3.67[a]	66.10±5.50[a]	23.81±6.54[a]
7.5	167	76.20±1.30[a]	69.80±6.12[a]	58.72±6.19[a]	25.89±10.24[a]
15	194	82.85±1.05[b]	76.26±3.37[a]	69.36±3.48[a]	44.83±5.76[b]
30	172	66.95±5.06[a]	69.20±5.02[a]	64.50±2.26[a]	18.69±5.86[a]
150	206	56.68±5.71[c]	65.25±6.49[a]	55.94±8.43[a]	17.85±8.98[a]
300	160	45.34±4.38[d]	60.28±4.23[a]	40.84±4.56[b]	9.26±5.61[c]

Data are presented as the mean ± standard error of the mean (SEM). Percentages of oocyte maturation, 4-cell embryos, and blastocysts were calculated from cultured oocytes, 2-cell embryos, and 4-cell embryos, respectively. For each parameter (maturation rate, cleavage rate, 4-cell rate, blastocyst rate), values with different superscript letters differ significantly ($P < 0.05$).
